# MiR-433 and miR-127 Arise from Independent Overlapping Primary Transcripts Encoded by the miR-433-127 Locus

**DOI:** 10.1371/journal.pone.0003574

**Published:** 2008-10-30

**Authors:** Guisheng Song, Li Wang

**Affiliations:** Departments of Medicine and Oncological Sciences, Huntsman Cancer Institute, University of Utah, Salt Lake City, Utah, United States of America; Ecole Normale Supérieure de Lyon, France

## Abstract

MicroRNAs play significant roles in development, metabolism and carcinogenesis, however, limited information is available about their primary transcripts and the transcriptional regulation of the microRNA genes. We report here the cloning of two primary miRNAs (pri-miR-433 and pri-miR-127) encoded by the miR-433-127 locus. Using both database mining and experimental methods, we isolated the full-length primary transcripts of the mouse miR-433 and miR-127 and demonstrated that they overlapped in a 5′-3′ unidirectional way. These two miRNA genes are expressed in a compact space by using overlapping coding regions. This is the first report to identify an economical structure for miR-433 and miR-127 genes, which may be a novel way of miRNA gene to maximize the genetic information in order to fit the complex physiological function of mammalian organism.

## Introduction

MicroRNAs (miRNAs or miR) are ∼22 nt small RNA molecules that regulate expression of target genes frequently by posttranscriptional repression mechanisms [1]. Mature miRNAs are typically generated via a two-step processing pathway. Primary miRNAs (pri-miR) transcribed by polymerase II or III are processed by the nuclear enzyme Drosha to produce precursor-miRNAs (pre-miR), which are then exported into the cytoplasm and further processed into mature miRNAs by Dicer [2]–[4]. Many studies are focusing on the function of miRNAs in physiological regulation and human diseases [5], [6], as well as identifying new miRNAs [7], [8]. However, little is known about the genomic structure of miRNA genes and their expression regulation. In this study, we set up a bioinformatics method to mine the potential primary transcript of miRNAs by EST extention. After bioinformatics analysis, we isolated the full-length primary transcripts of miR-433 and miR-127 and showed that they overlap in a 5′-3′ unidirectional way. These two miRNA genes are expressed in a compact space by using overlapping coding regions. This study suggests an economical gene structure for miR-433 and miR-127, which may be a novel way of miRNA gene to maximize the genetics information in order to fit the complex physiological function of mammalian organism.

## Results

### Database mining and EST extension

**Figure 1:**
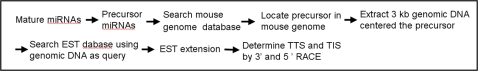
Schematic representation of the database mining for miRNA primary transcripts. MiRNA precursor sequences were used as a query to search the mouse genome database. After locating the miRNA precursor in the genome, a 3 kb sequence centered miRNA precursor was extracted and used as a query to search EST and non-coding RNA databases. After filtering the Blast hits, the EST surrounding the miR-precursor was used as template to design RACE primers to identify the transcriptional initiation site (TIS) and transcriptional termination site (TTS).

It is widely accepted that many of the miRNAs are transcribed by RNA polymerase II [2]. The primary transcripts of miRNAs (pri-miRNAs) contain 5′-m^7^G cap structures as well as 3′-end poly (A) tails, which are unique properties of class II gene transcripts. This implies that primary transcripts of some miRNAs could appear as expressed sequence tags (EST) or non-coding RNAs (ncRNAs). With the development of transcriptomics, many ESTs and ncRNAs are available in databases. Because pre-miR sequences are so short, the possibility that the precursor sequence is included in an EST sequence is low. As a step toward future miNomic (miR genomic) studies, especially the regulation of miRNA expression, we set out to use a computational approach to identify primary transcripts of miRNAs by EST extension (Figure 1).

The precursor sequences of miRNAs downloaded from the Sanger Institute (miRBase, 9) were used as a query in a BLASTN search of the mouse genome database to identify the location of miRNA precursors in the mouse chromosome. A 3 kb genomic sequence centered in the miRNA precursor was then extracted and used as a query to search the mouse EST and ncRNA database by local Blasting. After filtering the Blasting hits, we found that EST AK018276.1 (GenBank Accession number) includes miR-433 which is close to miR-127. Sequence analysis of AK018276.1 and its surrounding region in the chromosome showed that this EST stopped at the 6^th^ nucleotide of pre-miR-127. Based on this result, we hypothesized that EST AK018276.1 did not represent a full length cDNA. However, this sequence information allowed us to design specific primers for 5′RACE and 3′RACE to identify the 5′ transcriptional initiation site (TIS) and 3′ transcriptional termination site (TTS) for the miR-433-127 gene.

### Determine transcriptional initiation and termination sites of pri-miR-433 and pri-miR-127 by RACE

**Figure 2:**
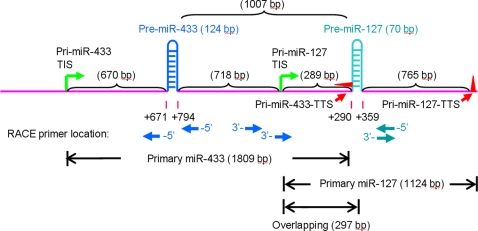
Complete genomic structure of miR-433-127 gene cluster. Chromosomal localization of miR-433 and miR-127 primary transcripts. Genes for miR-433 and miR-127 are located on chromosome 12 on the positive strand. The green arrows represent the TIS of the two overlapping transcripts, and the red triangles and arrows represent the TTS sites. The navy and teal arrows indicate the location of RACE primers used to clone pri-miR-344 and pri-miR-127, respectively. The length for the miR-433 and miR-127 gene is 1,842 bp and 1,124 bp, respectively. The TIS of pri-miR-127 is located within the 3′-end of pri-miR-433 and the pri-miR-433 transcript contains the promoter region of the miR-127 gene. These two transcripts share an overlapping region with a length of 297nt. The distance between the two precursor coding regions for miR-433 and 127 is 1,007 bp.

**Figure 3:**
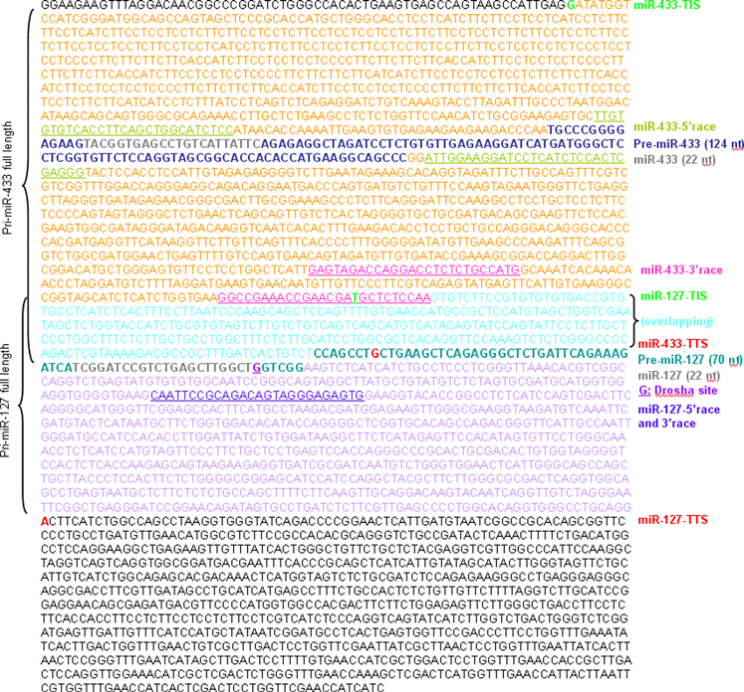
Complete genomic sequences of the miR-433-127 gene cluster. Full length sequences for pri-miR-433 and pri-miR-127 primary transcripts are presented. RACE primer sequences: underlined; TIS, transcriptional initiation site; TTS, transcriptional termination site. The color in word description matches with the color of the gene sequences.

**Figure 4:**
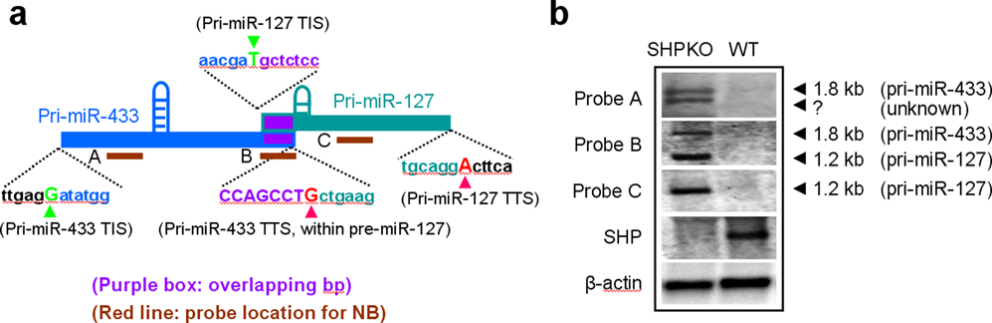
(a) Schematic of the miR-433 and miR-127 overlapping transcripts. The purple box shows the overlapping region of the miR-433 gene and miR-127 gene in a unidirectional manner. The bold red lines indicate the location of probes used for Northern blots (NB). The green and red letters and arrowheads point to the TIS and TTS of pri-miR-433 and pri-miR-127, respectively. Pri-miR-433 stops at the 8^th^ nucleotide of miR-127 precursor (pre-miR-127). (b) Northern blots of pri-miR-433 and pri-miR-127 overlapping transcripts. Probes A, B and C are designed with sequences complementary to the unique sequences of pri-miR-433, overlapping region of pri-miR-433 and pri-miR-127, and pri-miR-127, respectively. Total RNAs, which did not contain miRNAs, were isolated from livers of wild type (WT) and nuclear receptor small heterodimer partner SHP-knockout (SHPKO) mice. The transcript of pri-miR-433 with predicted length of ∼1.8 kb was detected using probe A in SHPKO mice. A smaller band was also detected by probe A, indicating an unknown transcript. The expression of pri-miR-127 (∼1.2 kb) was verified by a Northern blot using probe C in SHPKO mice. Two transcripts were detected by probe B with predicted size of pri-miR-433 and pri-miR-127, respectively. Both the primary pri-miR-433 and pri-miR-127 were significantly enriched in SHPKO mice. The same blot was used for probes A, B and C, after careful and complete washing and stripping following each hybridization.

Due to the lack of an open reading frame (ORF) or other features required by current gene recognition algorithms, it has been proposed that most of the miRNAs are expressed from independent transcription units [10]. This is particularly evident in *C. elegans* in which some ncRNA genes located within intronic regions are usually expressed from independent transcription units [11]. We hypothesized that the miR-433 and miR-127 genes may use overlapping genomic regions, because the chromosomal location of miR-433 and miR-127 was only ∼1 kb apart (Table S1). The 5′- and 3′-terminal nucleotides of pri-miR-433 were determined by 5′ and 3′RACE, respectively, which mapped a single TIS 670 bp upstream of pre-miR-433 and a single TTS 1,115 bp downstream of pre-miR-433 (Figure 2, navy primers; Figure 3 and Figure S1a and Figure S2). This predicted that the full length pri-miR-433 had a size of 1,809 nt. Using a strategy similar to that described for pri-miR-433, the TIS of pri-miR-127 was determined to be 289 nt upstream of the pre-miR-127, which located in the coding region of miR-433 primary transcript (Figure 2, teal primers; Figure 3 and Figure S1b). The 3′-terminal sequence of pri-miR-127 was also determined to be in complete agreement with the size predicted for the full length pri-miR-127 (1,124 bp). Non-conserved sequences were observed surrounding both of the TIS sites (G in bold green for pri-miR-433 and T in bold green for pri-miR-127) (Figure 4a). No sequences resembling a consensus TATA box (TATAAA) were identified near the TIS sites of pri-miR-433 and pri-miR-127, an observation that was similar to the human miR-34a gene [12]. Interestingly, our data did not identify a heterogeneous transcription start site, as present in other miRNAs [2], [12]. Remarkably, none of the two transcripts originating from this locus displayed the consensus AAUAAA polyadenylation signal, which is different from the reported human pri-miR-21 and pri-miR-34a [2], [12]. Lack of polyadenylation signal is uncommon in eukaryotic genes, but it has been reported for other overlapping protein coding genes [13]. Our data suggest that this phenomenon may also exist for the non-protein coding miRNA genes. It is not clear how the 3′ processing of these miRNAs occurs, but it may involve premature termination of transcription.

The RACE primers were designed within the cloned miRNA RACE products and multiple individual clones were sequenced to verify the finding of the overlapping miR-433/127 genes. For instance, the nested 5′RACE primers for pri-miR-433 were designed right after (includes pre-miR-433) and before (excludes pre-miR-433) pre-miR-433 and sequencing confirmed the overlapping and prolongation of the 2^nd^ PCR from the 1^st^ PCR. In addition, the 3′RACE amplified region of pri-miR-433 using 3′RACE primers was also an integral part of the 5′RACE of pri-miR-127, providing evidence for an overlapping region of the 3′-end of the miR-433 gene within the 5′-end of the miR-127 gene. Moreover, both the 1^st^ and 2^nd^ 3′RACE pri-miR-433 stopped at the predicted TTS but failed to amplify the full length pre-miR-127. Finally, a contiguous transcript including both miR-433 and miR-127 could not be detected using miR-127 5′RACE, which stopped at the predicted pri-miR-127 TIS. Although the possibility may occur for an internal priming of the oligo(dT) RACE primer to an adenosine rich genomic stretch and the actual polyadenylation site may be found further downstream, the Northern blots described below strongly suggested that these two pri-miRNAs were overlapped. Taken together, pri-miR-433 and pri-miR-127 were arranged in a tail-to-head orientation that were transcribed in the same direction, and shared a 297 bp overlapping region (the complete sequence for miR-433-127 gene cluster was deposited in GenBank, Accession number EU499599).

### Northern blot analysis of pri-miR-433 and pri-miR-127 primary transcript

Our recent study has identified the nuclear receptor small heterodimer partner (SHP) as a negative regulator of miR-433 and miR-127 gene transcription [14]. Both miR-433 and miR-127 exhibit increased expression in SHPKO mice as compared to the wild-type (WT) mice. Interestingly, the fold increase of miR-433 (∼4 fold) and miR-127 (∼40 fold) in SHPKO mice appears to be drastically different. Thus, we predict that the pri-miR-433 and pri-miR-127 would also be significantly increased but differentially expressed in SHPKO mice. Northern blots were used to further verify the length and the overlapping pri-miR-433 and pri-miR-127 using total RNAs isolated from WT and SHPKO mice. The expression of pri-miR-433 and pri-miR-127 transcripts was observed to be strongly upregulated in the livers of SHPKO mice than in WT mice (Figure 4b). Northern blots (Figure 4a) using probe A (uniquely complementary to the 5′-terminus of pre-miR-433) identified an RNA species similar in size to pri-miR-433 (Figure 4b) in SHPKO mice. In contrast to probe A, probe C (uniquely complementary to the 3′-terminal end of pre-miR-127) identified an RNA species with a size similar to that of pri-miR-127. Probe B, whose sequence is complementary to the overlapping pri-miR-433 and pri-miR-127 coding sequence, hybridized to two RNA species corresponding to the size of pri-miR-433 and pri-miR-127, respectively. Thus, we concluded that both RNAs represented the predicted pri-miR-433 and pri-miR-127. Because miRNAs were not included in the total RNA population during RNA isolation, we did not observe multiple hybridization bands that may result from pri-miRNA processing. Instead, clear and sharp bands representing both primary transcripts were observed. The above results provided further evidence that these two pri-miRNAs produced from the miR-433-127 locus were transcribed from two separate genes that shared overlapping region. The basal expression of pri-miR-127 appeared to be several fold higher than pri-miR-433, consistent with the higher expression level of miR-127 as compared to miR-433 [14]. These observations suggest that the transcription of miRNA genes can be regulated in a subtle and interactive manner even though the miRNA genes are packaged in a compressed way.

## Discussion

Overlapping genes are defined as adjacent genes that overlap partially or entirely. Typically, two overlapping genes are encoded on opposite strands of genomic DNA. Overlapping sense/antisense genes orientated in a tail-to-tail manner, usually involves only the 3′UTRs and form the majority of “overlapping” gene pairs in the mammalian genomes. In mammals, several overlapping genes have been identified, including two neuropeptide Y genes, the INK4A/ARF locus and the VLCAD/DLG4 genes [15]–[17]. However, for miRNA genes, this is the first time to discover and prove that they are transcribed by using overlapped region. Among viruses, overlapping genes are employed to compress the genome to a small packageable size [18]. Overlapping gene pairs extracted from complete bacterial genome sequences are classified into three directional patterns, namely, ‘convergent’ (→←), ‘unidirectional’ (→→), and ‘divergent’ (← →) [19]. The overlapping miR-433/127 gene pair represents a unidirectional pattern.

A recent report assumed that the human miR-127 and miR-433 may be spliced from a large transcript including the whole cluster [20]. However, the primary transcript has not been cloned and this assumption has not been verified. The data presented here suggest that this may not be the case in mice. Cloning of the human primary transcript encoding miR-433 and miR-127 will certainly determine if a similar overlapping gene structure exists in other mammalian species.

It is noted that probe A also hybridized to a smaller transcript. The nature of the transcript is currently unknown. One possibility is that it may result from an alternate start site. However, our RACE data did not identify a heterogeneous transcript start site. Recently we report that a cluster of miRNAs is co-induced in nuclear receptor small heterodimer partner (SHP) knockout mice [14]. MiR-431 is located about 1 kb upstream from miR-433. It is possible that the genes encoding miR-431 and miR-433 may also overlap and that this small transcript may represent pri-miR-431. We are undertaking the effort to identify the transcriptional initiation and termination sites for pri-miR-431 and determine if pri-miR-431 overlaps with pri-miR-433.

One interesting question is whether the two miRNAs arise from completely independent primary transcripts or whether the longer primary transcript is responsible for generating both miRNAs and a short one encodes miR-127. The results from this study and our published data [14] suggest that the latter seems unlikely the case. Our study shows that the coupled miR-433 and miR-127 genes are transcribed from independent promoters by using overlapping genomic regions [14]. The 3′-coding region of pri-miR-433 serves as the promoter region of pri-miR-127. This is supported by the observation that both primary and mature miR-433 and miR-127 exhibit different fold induction in SHPKO mice, suggesting a differential and independent regulation of gene expression.

The miRNA cluster containing miR-433 and miR-127 is located within the imprinted Dlk1/Gtl2 region that are only expressed from the maternal chromosome [21]. MiR-127 and miR-136 are reported to be processed from a transcript (*antiPeg11*) that is antisense to Rtl1/Peg11, a paternally expressed intronless gene [22], [23]. A recent study shows that miR-433 is also processed from *antiPeg11* [24]. These studies established an interesting link for miRNA-mediated regulation involving imprinted genes. Despite these important findings, how the genes encoding the miRNAs are assembled and how the miRNAs are processed from their primary transcripts remain unknown. Our study for the first time elucidates an overlapping gene structure for miR-433 and miR-127. It is possible that such an overlapping gene structure may also exist for other miRNAs, such as miR-431 and miR-433. Determining transcriptional initiation and termination sites for each miRNA will allow to determine if the multiple transcripts of miRNAs are produced from overlapping genes or polycistronic effect may play a role.

Although the mammalian genome is about 800 fold larger than the *E. coli* genome, the gene expression regulation network is unexpectedly more complex. Given the relatively large size of the mammalian genome, the discovery of overlapping coding regions for miRNA genes raises several interesting questions. For example, is this simply due to the conservation of an ancient pri-miRNA gene pair structure or is there a purpose for this overlapping gene structure that facilitates a complex and interactive physiological regulation of mammalian miRNA expression? Nevertheless, the economical structure of the miR-433/127 locus is a novel finding regarding the structure of mammalian miRNA genes, which may be a novel way of miRNA gene to maximize the genetics information in order to fit the complex physiological function of mammalian organism. Determining the detailed mechanisms how miR-433 and miR-127 are transcribed and regulated should provide insight into why such an unusual gene structure exists for these miRNAs.

## Methods

### Database mining of primary transcripts of miRNAs and EST extension

Fifty five miRNA precursor sequences on mouse chromosome 12 were downloaded from the Sanger Institute (http://microrna.sanger.ac.uk/sequences/). The BLASTN search of the mouse genome was done online. The mouse EST and ncRNA database were downloaded from GenBank. A 3 kb genomic sequence centered in the miRNA precursor was extracted manually and used as query to Blast in the EST and ncRNA database with the command “Megablast -e 1e-100 -F “m L” -D 3”. The Blast Packages (v2.2.10) were downloaded from the NCBI website (ftp://ftp.ncbi.nlm.nih.gov.blast/executables/). The ESTs with the opposite transcription direction compared to query were removed from the Blast hits. The hits with an aligned length less than 95 of their original length were filtered out. After filtering, the ESTs matched to the −2 kb to 2 kb flanking regions of miRNA precursors were selected for further analysis.

### RACE mapping of miRNA primary transcript

Total liver RNA was isolated using RNeasy Mini Kit (Qiagen, Valencia CA) and mRNA was isolated using Oligotex Direct mRNA Mini Kit (Qiagen, Valencia CA). The GeneRacer Kit (Invitrogen, California USA) was used to map the transcription initiation site and polyA signal site of primary transcripts. The first strand cDNA was synthesized at 65°C with Thermo-X™ reverse transcriptase using 2 µg of mRNA, followed by a polymerase chain reaction with 95°C denaturation step and then 45 cycles of touchdown annealing temperature. Primer sequences are available upon request.

### Northern blotting analysis

Total RNA was extracted from livers of two-month old wild type and SHP knockout mouse using Trizol Reagent (Invitrogen). RNA probes of the mouse beta-actin, SHP, probe A, B and C were labeled with biotin-11-UTP using the MAXIscript® In Vitro Transcription Kit (Ambion). 2 µg of biotin-labeled RNA probe was used to hybridize using NorthernMax (Ambion). In brief, 30 µg of total RNA was fractionated on a denaturing agarose gel, transferred to Nylon ^N+^ membranes by a capillary method and fixed by ultraviolet cross-linking. Membranes were probed with biotin-labelled RNA. Prehybridization and hybridizations were performed in ULTRAhyb® Ultrasensitive Hybridization Solution at 65°C. The labeled probes were heated for 3 min at 70°C before adding the prehybridization solution. After hybridization, the membranes were washed at low stringency in 2×SSC, 0.1% SDS at room temperature twice for 5 min or at high stringency in 0.1 SSC, 0.1% SDS at 65°C twice for 15 min. A chemiluminescent procedure BrightStar® BioDetect™ Kit (Ambion) was used to visualize the probes.

## Supporting Information

Table S1 Chromosomal location of the miR-433 and miR-127.
(0.17 MB PDF)

Figure S1 Determining the transcriptional initiation site (TIS) by 5′-RACE and transcriptional termination site (TTS) by 3′-RACE for pri-miR-433 and pri-miR-127 gene. Total liver RNA was isolated using RNeasy Mini Kit (Qiagen, Valencia CA) and mRNA was isolated using Oligotex Direct mRNA Mini Kit (Qiagen, Valencia CA). The GeneRacer Kit (Invitrogen, California USA) was used to map the transcription initiation site and polyA signal site of primary transcripts.
(2.30 MB TIF)

Figure S2 Chromatogram of 3′ RACE sequences of pri-miR-433 primary transcript. The transcriptional termination site of pri-miR-433 (G) is indicated by pink arrow. Poly-A-tail follows immediately after “G”.
(2.30 MB TIF)
