# Supplementary material for: MiR-433 and miR-127 Arise from Independent Overlapping Primary Transcripts Encoded by the miR-433-127 Locus
Source: PLoS One. 2008 Oct 30;3(10):e3574. doi: 10.1371/journal.pone.0003574 (PMC2570487; doi:10.1371/journal.pone.0003574)
Supplement: Table S1 — Chromosomal location of the miR-433 and miR-127. (0.17 MB PDF) [file pone.0003574.s001.pdf]

Genomic location of microRNA-433-127 cluster on mouse chromosome 12

miRNA	Chr	miRNA Strand	miRNA Start	miRNA End
mmu-miR-433	12	NA / +	110039521	110039645
mmu-miR-127	12	NA / +	110040652	110040722

Song et al., Table S1
