# Supplementary figures and images for: MiR-433 and miR-127 Arise from Independent Overlapping Primary Transcripts Encoded by the miR-433-127 Locus
Source: PLoS One. 2008 Oct 30;3(10):e3574. doi: 10.1371/journal.pone.0003574 (PMC2570487; doi:10.1371/journal.pone.0003574)

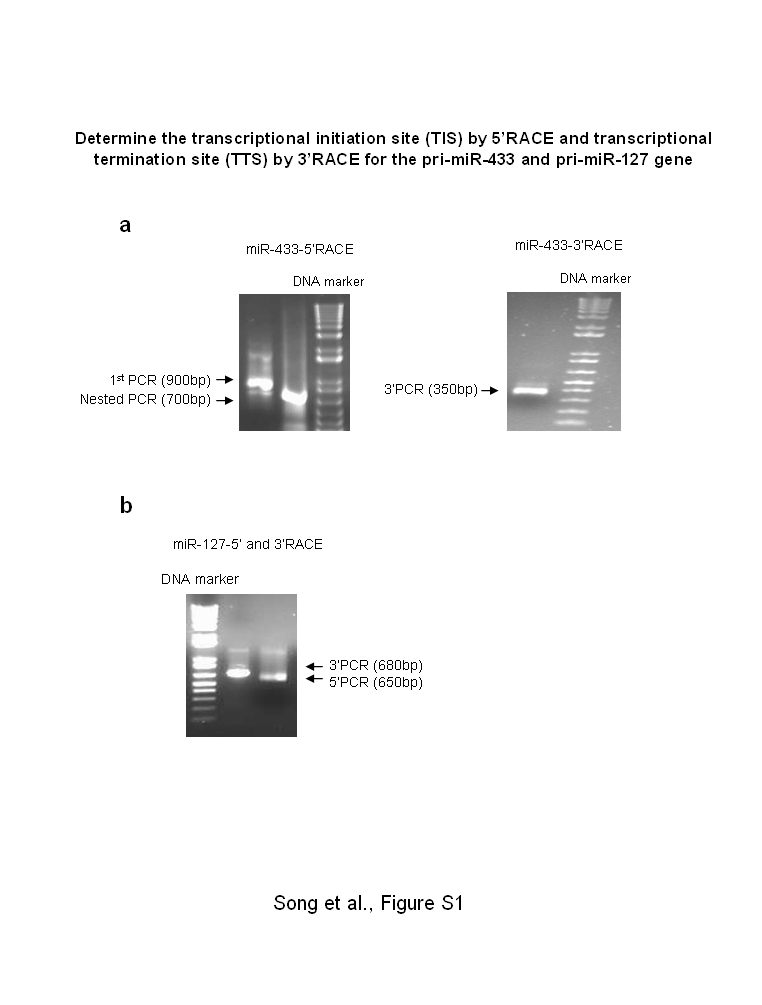

Supplement: Figure S1 — Determining the transcriptional initiation site (TIS) by 5′-RACE and transcriptional termination site (TTS) by 3′-RACE for pri-miR-433 and pri-miR-127 gene. Total liver RNA was isolated using RNeasy Mini Kit (Qiagen, Valencia CA) and mRNA was isolated using Oligotex Direct mRNA Mini Kit (Qiagen, Valencia CA). The GeneRacer Kit (Invitrogen, California USA) was used to map the transcription initiation site and polyA signal site of primary transcripts. (2.30 MB TIF) [file pone.0003574.s002.tif]

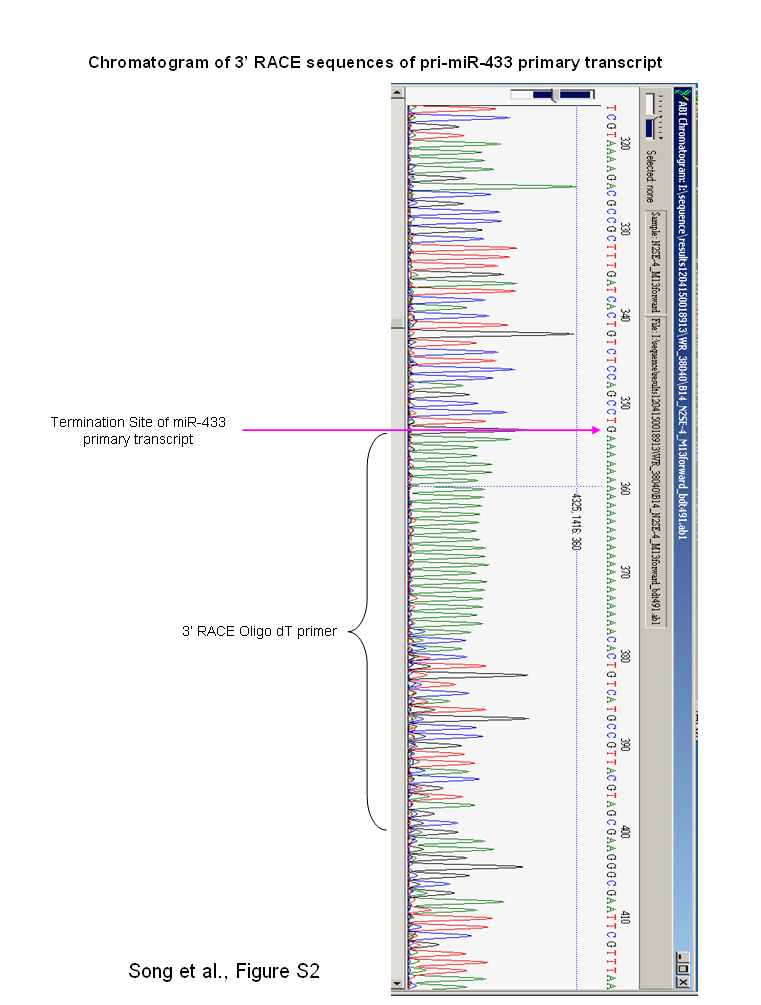

Supplement: Figure S2 — Chromatogram of 3′ RACE sequences of pri-miR-433 primary transcript. The transcriptional termination site of pri-miR-433 (G) is indicated by pink arrow. Poly-A-tail follows immediately after “G”. (2.30 MB TIF) [file pone.0003574.s003.tif]
